# Clinical comparison of HMPV and RSV infections in hospitalised Malaysian children: A propensity score matched study

**DOI:** 10.1111/crj.13747

**Published:** 2024-03-26

**Authors:** David Chun‐Ern Ng, Chuin‐Hen Liew, Kah Kee Tan, Elida Hanan binti Awang, Farah Nuruliayana binti Ahmad Nazri, Asuwani K. Tamil Maran, Vishnu Arvindran a/l Chandra Mohan, Durairaaj Ramachandran, Michelle Chok, Cheah Hooi Teh, Airena Mohamad Nor, Suhaila bt Baharuddin, Erwin Jiayuan Khoo

**Affiliations:** ^1^ Hospital Tuanku Ja'afar Ministry of Health Malaysia Seremban Malaysia; ^2^ Hospital Tuanku Ampuan Najihah Ministry of Health Malaysia Kuala Pilah Malaysia; ^3^ Department of Paediatrics Perdana University Seremban Clinical Academic Center Seremban Malaysia; ^4^ Department of Paediatrics International Medical University Kuala Lumpur Malaysia; ^5^ Department of Global Health and Social Medicine Harvard Medical School Boston Massachusetts USA

**Keywords:** hMPV, paediatric, predictors, RSV, severity

## Abstract

**Introduction:**

Human metapneumovirus (hMPV) and respiratory syncytial virus (RSV) are significant contributors to the burden of acute respiratory infections in children, but data on hMPV from Southeast Asia are limited despite its potential for serious disease. This study aimed to compare the clinical presentation, resource utilisation and outcomes between hMPV and RSV infections in hospitalised Malaysian children.

**Methods:**

This retrospective, observational study included children aged ≤12 years old hospitalised with hMPV or RSV, confirmed via direct fluorescent antibody (DFA) methods, between 1 July to 30 October 2022 at Hospital Tuanku Ja'afar Seremban, Malaysia. Demographic, clinical presentation, resource utilisation and outcome data were analysed. Propensity score matching was used to balance cohorts based on key demographic and clinical characteristics.

**Results:**

This study included 192 patients, comprising 112 with hMPV and 80 with RSV. hMPV patients were older (median age 20.5 vs. 9.4 months, *p <* 0.001) and had a higher incidence of comorbidities (24.1% vs. 7.5%, *p =* 0.003). Fever was more common in the hMPV group (97.3% vs. 73.8%, *p <* 0.001), but the other clinical manifestations were similar. Postmatching analysis showed higher corticosteroid use in the hMPV group (*p =* 0.01). No significant differences were observed in the use of other resources, PICU admissions, duration of hospitalisation or mortality rates between both groups.

**Conclusion:**

hMPV and RSV infections in children share similar clinical manifestations and outcomes, with hMPV affecting older children and showing higher corticosteroid usage. These findings emphasise the need for equal clinical vigilance for both hMPV and RSV in paediatric respiratory infections.

## Graphical abstract

In this study comparing human metapneumovirus (hMPV) and respiratory syncytial virus (RSV) infections in hospitalised children, hMPV patients were older and exhibited more comorbidities and fever compared with RSV patients. Propensity score matching analysis indicated higher corticosteroid usage in hMPV cases. However, hospital resource utilisation and clinical outcomes were similar between both groups, underscoring the need for equal clinical vigilance for both viruses.

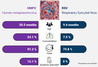

## 1 INTRODUCTION

Human metapneumovirus (hMPV) and respiratory syncytial virus (RSV), members of the *Paramyxoviridae* family, are major causes of acute respiratory infections in children worldwide. These pathogens can present with a spectrum of illness in children, ranging from mild disease requiring only outpatient treatment to severe lower respiratory tract infections that may potentially require paediatric intensive care unit (PICU) admission.

Distinguishing hMPV from RSV solely based on clinical signs is challenging due to their similar presentations and overlapping seasonal distribution patterns.^1^, ^2^ However, the severity and clinical outcomes of these infections are variable in the literature, particularly concerning healthcare resource utilisation and patient management.^3^, ^4^, ^5^, ^6^, ^7^

The role of RSV as a leading cause of acute respiratory illnesses in children has been well‐documented globally. In contrast, hMPV, which was identified more recently, remains less extensively studied, despite increasing evidence that it can also lead to significant morbidity and economic burden.^8^, ^9^ Studies on paediatric hMPV from Southeast Asia are limited, yet vital, as regional variations in demographics, comorbidities, healthcare access and other factors could significantly influence the severity and clinical outcomes.

Hence, our study focuses on the Malaysian paediatric context, aiming to provide a detailed comparison of the clinical presentation, resource utilisation and outcomes of hMPV and RSV infections in hospitalised children. The findings of this study would enhance the understanding of the differential impact of hMPV and RSV on resource utilisation and guide healthcare providers in optimising hospital resources for managing these respiratory infections in children.

## 2 MATERIALS AND METHODS

### 2.1 Design and setting

This study was a hospital‐based, retrospective study of children aged ≤12 years hospitalised with hMPV or RSV infections between 1 July 2022 and 30 October 2022. The study was conducted at Hospital Tuanku Ja'afar Seremban, Negeri Sembilan, Malaysia. The hospital is the only tertiary referral centre with PICUfacilities in the state, serving approximately 1 200 000 people, including 260 000 children ≤12 years.

### 2.2 Data collection and study definitions

We included all children aged ≤12 years old who were hospitalised with laboratory confirmed hMPV and RSV infections during the study period. Children with codetection of multiple respiratory viruses and bacterial coinfections were excluded from the analysis to avoid any potential confounding on the clinical presentation and outcome.

The patient's medical records in the hospital's electronic database were reviewed, and data were extracted using a standardised data collection form. Information retrieved included demographic characteristics, existing comorbidities, clinical and laboratory parameters and outcome measures such as PICU admission, duration of hospital stay and mortality rates. In addition, the utilisation of hospital resources such as intravenous (IV) fluids, bronchodilators, corticosteroids, antibiotics and respiratory support was recorded.

All symptoms and signs were collected at the time of presentation. Fever was defined as temperature ≥37.5°C. Duration of illness was calculated from the first symptom onset. A potential source of infection with an adult or child was specified based on the age and nature of contact, as defined previously.^10^ When multiple family members were symptomatic, the first family member who became symptomatic was regarded as the sick contact. Adventitious breath sounds were defined as the presence of either crackles, wheeze or rhonchi on lung auscultation. Severe disease was defined as patients who required admission to the PICU for advanced respiratory support, inotropic support or intensive monitoring.^10^, ^11^ Patients were categorised according to clinical phenotypes on presentation such as upper respiratory tract infection, lower respiratory tract infection, asthma exacerbation and febrile seizures, which were described previously.^12^ The decision to prescribe systemic corticosteroids was at the discretion of the attending clinician, taking into account the severity of respiratory symptoms, the presence of underlying asthma or hyperactive airways and the patient's overall response to initial treatment.

### 2.3 Laboratory methods

A nasopharyngeal aspirate (or endotracheal aspirate if ventilated) was obtained from each patient who presented with fever and/or cough, regardless of the presenting clinical phenotype. This sampling was performed within the initial 24 h of their hospital stay. The collected samples were tested using direct fluorescent antibody (DFA) assay for antigen detection. We utilised the D3 Ultra™ DFA Respiratory Virus Screening and Identification Kit (Diagnositic Hybrids, Inc., USA), which is capable of detecting RSV, hMPV, adenovirus, influenza A, influenza B and parainfluenza types 1–3.

### 2.4 Statistical analysis

Categorical variables were presented as frequency and percentage (%), and comparisons were conducted using the Chi‐squared test or Fisher's exact test, as appropriate. Continuous variables in this study, which displayed a skewed distribution as evidenced by the Kolmogorov–Smirnov test, were presented as median and interquartile range (IQR). The nonparametric two‐tailed Mann–Whitney *U*‐test was used for analysis of continuous variables. A *p*‐value <0.05 was considered statistically significant. All analyses were performed using SPSS version 26.0 (IBM Corp, Armonk, NY, USA).

To compare the resource utilisation and outcomes between hMPV and RSV patients, we conducted propensity score matching with the aim of reducing selection bias. Patients were matched in a 1:1 ratio using nearest neighbour matching with a calliper width of 0.2 standard deviations. The matching focused on key parameters with a standardised mean difference (SMD) greater than 0.2, including age, sex, comorbidities, presence of fever and temperature on arrival. Propensity scores were calculated and matched using the Rcmdr Plug‐In Package in R (version 4.2.1). To assess balance postmatching, the effect size was expressed as Cohen's *d* (SMD), categorising values as trivial (SMD < 0.2), small (SMD 0.2 and 0.49), moderate (0.5 to 0.79) and large (SMD ≥ 0.8).^13^

Outliers were identified in seven variables, with the highest outlier percentage being 4.4% (supporting information Table S1). These outliers were acknowledged as natural outliers and were retained for analysis. Seven variables with missing values were encountered. We addressed these missing values through multiple imputations, except for variables where more than 50% of the data was missing, which were excluded from the analysis. Further details on data management and handling of missing data are provided in supporting information Table S2.

## 3 RESULTS

During the study period, 206 children hospitalised with a positive respiratory viral test for RSV or hMPV were identified. After excluding 14 patients due to viral and bacterial coinfection, the study population comprised 192 patients: 112 with hMPV infection and 80 with RSV infection.

Table 1 presents the demographic characteristics. The hMPV‐infected patients were significantly older, with a median age of 20.5 months, compared with the RSV group's median age of 9.4 months (*p <* 0.001). Additionally, the presence of comorbidities was significantly higher in the hMPV group, with 24.1% having at least one comorbidity, compared with 7.5% in the RSV group (*p =* 0.003). Notably, no patients in this study received Palivizumab.

**TABLE 1:** Comparison of demographic and baseline characteristics of children hospitalised with hHMPV and RSV.

	hMPV (*n* = 112)	RSV (*n* = 80)	*p*‐value
Age in months, median (IQR)	20.5 (9.9–33.3)	9.4 (3.4–22.4)	<0.001^a^
Male sex	61 (54.5%)	52 (65.0%)	0.14^b^
Ethnicity			
Malay	105 (93.8%)	75 (93.8%)	1.00^c^
Chinese	2 (1.8%)	2 (2.5%)	
Indian	3 (2.7%)	2 (2.5%)	
Others	2 (1.8%)	1 (1.2%)	
Sick contact			
Child	53 (47.3%)	48 (60.0%)	0.17^b^
Adult	14 (12.5%)	10 (12.5%)	
Comorbidities^d^			
None	85 (75.9%)	74 (92.5%)	0.003^b^
Respiratory	12 (10.7%)	1 (1.2%)	0.01^b^
Cardiac	10 (8.9%)	2 (2.5%)	0.07^b^
Genetic	7 (6.3%)	0 (0%)	0.04^c^
Ex‐prematurity (if <2 years old)	6 (5.4%)	2 (2.5%)	0.47^c^
Neuromuscular	4 (3.6%)	1 (1.2%)	0.40^c^
Others	4 (3.6%)	1 (1.2%)	0.40^c^

Abbreviations: hMPV, human metapneumovirus; IQR, nterquartile range; RSV, respiratory syncytial virus.

^a^ Mann–Whitney test.

^b^ Chi‐square test.

^c^ Fisher exact test.

^d^ A patient may have more than 1 comorbidity.

Table 2 demonstrates the clinical features and laboratory investigation findings on admission. Fever was more commonly reported among hMPV patients (97.3% vs. 73.8%, *p <* 0.001), corresponding to the higher median temperature on arrival (38.1°C vs. 37.3°C, *p <* 0.001). Both groups predominantly presented with lower respiratory tract infection phenotypes (90.2% in hMPV cohort and 95.0% in RSV cohort, *p =* 0.22). Febrile seizures were equally low and not significantly different between the groups: 1.8% in hMPV and 2.5% in RSV (*p =* 1.00). Laboratory investigations revealed that both hMPV and RSV had comparable total white cell and absolute lymphocyte counts.

**TABLE 2:** Comparison of clinical and laboratory characteristics of children hospitalised with hHMPV and RSV.

	hMPV (*n* = 112)	RSV (*n* = 80)	*p*‐value
Day of illness on presentation, median (days)	4 (3–5)	3 (3–5)	0.14^a^
Fever	109 (97.3%)	59 (73.8%)	<0.001^b^
Temperature on arrival in **°**C, median (IQR)	38.1 (37.2–39.0)	37.3 (37.0–38.1)	<0.001^a^
Cough	108 (96.4%)	80 (100%)	0.14^c^
Rhinorrhea	78 (69.6%)	61 (76.2%)	0.31^b^
Seizures	3 (2.7%)	5 (6.2%)	0.28^c^
Vomiting	9 (8.0%)	4 (5.0%)	0.41^b^
Diarrhoea	13 (11.6%)	9 (11.2%)	0.94^b^
Tachypnea	103 (92.0%)	71 (88.8%)	0.45^b^
Chest recessions	97 (86.6%)	70 (87.5%)	0.86^b^
Spo2 < 92%	10 (8.9%)	4 (5.0%)	0.30^b^
Adventitious breath sounds	98 (87.5%)	70 (87.5%)	1.00^b^
Rashes	2 (1.8%)	0 (0.0%)	0.51^c^
Clinical phenotype			
Lower respiratory tract infection	101 (90.2%)	76 (95.0%)	0.22^b^
Exacerbation of asthma	4 (3.6%)	0 (0.0%)	0.14^c^
Upper respiratory tract infection	2 (1.8%)	1 (1.2%)	1.00^c^
Febrile seizures	2 (1.8%)	2 (2.5%)	1.00^c^
Total white cell count × 10^9^/L, median (IQR)	10.2 (7.9–13.4)	11.8 (8.3–14.8)	0.26^a^
Absolute lymphocyte count × 10^9^/L, median (IQR)	3.4 (2.2–5.0)	4.2 (2.5–6.2)	0.05^a^
Platelet count x 10^9^/L, median (IQR)	313 (252–391)	356 (298–489)	0.01^a^

Abbreviations: hMPV, human metapneumovirus; IQR, interquartile range; RSV, respiratory syncytial virus.

^a^ Mann–Whitney test.

^b^ Chi‐square test.

^c^ Fisher exact test.

Table 3 compares the characteristics of hMPV and RSV patients before and after propensity score matching. The initial unmatched cohort consisted of 112 hMPV and 80 RSV patients, while the matched cohort had 61 patients in each group. The patients were matched to balance the differences between the age, sex, presence of comorbidities, fever, and temperature on arrival. Postmatching, the median age of hMPV patients was 25.8 months (IQR 12.5–36.9) and for RSV patients was 14.5 months (IQR 7.5–26.0), with the SMD reducing from 0.48 to 0.41. The differences in sex and comorbidities were effectively balanced postmatching with SMD reducing from 0.22 to 0.08 and from 0.47 to 0.06, respectively. The matching process also eliminated the differences in the proportion of patients with fever and temperature on arrival between both groups.

**TABLE 3:** Comparison of demographic and clinical characteristics between hMPV and RSV patients before and after propensity score matching.

	Unmatched comparison			Propensity matched comparison		
	hMPV (*n* = 112)	RSV (*n* = 80)	SMD	hMPV (*n* = 61)	RSV (*n* = 61)	SMD
Age (months)	20.5 (9.9–33.3)	9.4 (3.4–22.4)	0.48	25.8 (12.5–36.9)	14.5 (7.5–26.0)	0.41
Male sex	61 (54.5%)	52 (65.0%)	0.22	59 (96.7%)	58 (95.1%)	0.08
Comorbidities	27 (24.1%)	6 (7.5%)	0.47	5 (8.2%)	6 (9.8%)	0.06
Fever	109 (97.3%)	59 (73.8%)	0.71	58 (95.1%)	58 (95.1%)	0
Temperature on arrival (°C)	38.1 (37.2–39.0)	37.3 (37.0–38.1)	0.62	37.6 (37.0–38.3)	37.5 (37.0–38.4)	0.04

Abbreviation: hMPV, human metapneumovirus; RSV, respiratory syncytial virus; SMD, standardised mean difference.

Table 4 compares the healthcare resource utilisation and clinical outcomes between hMPV and RSV patients before and after propensity score matching. In the unmatched cohort, hMPV patients demonstrated higher usage of intravenous fluids (58.9% vs. 36.3%) and corticosteroids (8.0% vs. 0%) compared with RSV patients, with SMDs of 0.47 and 0.42, respectively. Following propensity score matching, the differences in the utilisation of intravenous fluids narrowed, as indicated by a lower SMD of 0.17. However, the higher usage of systemic corticosteroids in the hMPV group persisted even after matching, with a SMD of 0.55. Other aspects of healthcare utilisation such as the use of empirical antibiotics, nebulised salbutamol, inotropic support and various forms of respiratory support showed trivial differences between the groups after matching, with SMDs close to or below 0.2. Similarly, the matched cohorts showed minimal differences in the median duration of oxygen therapy, length of hospitalisation, PICU admission rates and mortality between hMPV and RSV patients.

**TABLE 4:** Comparison of healthcare resource utilisation and clinical outcomes in hMPV and RSV patients before and after propensity score matching.

	Unmatched comparison			Propensity matched comparison		
	hMPV (*n* = 112)	RSV (*n* = 80)	SMD	hMPV (*n* = 61)	RSV (*n* = 61)	SMD
Intravenous fluids	66 (58.9%)	29 (36.2%)	0.47	30 (49.2%)	25 (41.0%)	0.17
Empirical antibiotics	62 (55.4%)	39 (48.8%)	0.13	32 (52.5%)	30 (49.2%)	0.07
Nebulised salbutamol	37 (33.0%)	20 (25.0%)	0.18	20 (32.8%)	15 (24.6%)	0.18
Systemic corticosteroids	9 (8.0%)	0 (0%)	0.42	8 (13.1%)	0 (0%)	0.55
Inotropic support	3 (2.7%)	0 (0%)	0.24	2 (3.3%)	0 (0%)	0.26
Respiratory support						
Low‐flow nasal cannula	48 (42.9%)	31 (38.8%)	0.08	30 (49.2%)	24 (39.3%)	0.20
High‐flow nasal cannula	28 (25.0%)	25 (31.2%)	0.14	14 (23.0%)	20 (32.8%)	0.22
Noninvasive ventilation	14 (12.5%)	9 (11.2%)	0.04	5 (8.2%)	3 (4.9%)	0.13
Mechanical ventilation	6 (5.4%)	2 (2.5%)	0.15	4 (6.6%)	2 (3.3%)	0.15
Median duration of oxygen therapy, days (IQR)	3.0 (2.0–4.0)	3.0 (2.0–5.0)	0.14^a^	2.0 (1.5–3.0)	3.0 (1.0–5.0)	0.22^b^
Median length of hospitalisation, days (IQR)	3.0 (2.0–4.0)	3.0 (2.0–5.0)	0.15	2.0 (1.5–3.0)	2.0 (2.0–4.5)	0.19
PICU admission	20 (17.9%)	12 (15.0%)	0.08	9.0 (14.8%)	6.0 (9.8%)	0.15
Mortality	1 (0.9%)	0 (0%)	0.13	1 (1.6%)	0	0.18

Abbreviations: hMPV, human metapneumovirus; IQR, interquartile range; RSV, respiratory syncytial virus; SMD, standardised mean difference.

^a^ Comparative analysis was performed on 96 versus 67 patients for HMPV and RSV groups.

^b^ Comparative analysis was performed on 53 versus 49 patients for HMPV and RSV groups.

## 4 DISCUSSION

In this study, we compared the differences in clinical presentation, resource utilisation and clinical outcomes between hMPV and RSV infections in hospitalised paediatric patients. We observed a notable difference in age distributions between these infections. Children admitted with hMPV infection were significantly older than those with RSV, consistent with previous studies.^14^, ^15^, ^16^ The age discrepancy is intriguing, considering that maternal antibodies for both viruses decline at roughly the same pace, reaching nadirs around 3 to 5 months.^17^, ^18^ One possible explanation for the age differences could be that hMPV requires a lower threshold of antibodies to confer protection.^17^ Therefore, the waning maternal antibodies might offer protection against hMPV for longer periods compared with RSV.

The clinical manifestations of hMPV and RSV were largely similar. However, fever was more frequently observed in patients with hMPV infection, a finding supported by previous studies.^19^, ^20^ Additionally, we identified a small subset of patients presenting with febrile seizures. Although previous studies have infrequently linked hMPV with febrile seizures,^21^, ^22^ our findings revealed a comparable proportion of febrile seizures in both the hMPV and RSV groups.

In comparing the outcomes between hMPV and RSV, previous studies often relied on unmatched cohorts, which may introduce confounding factors and selection bias.^14^, ^15^ To address this issue, we employed propensity score matching to create comparable patient groups with balanced key variables such as age, sex, comorbidities, fever and temperature on arrival. This approach was intended to reduce the likelihood that observed differences in treatment, and outcomes were influenced by demographic or clinical differences, rather than the viral infection itself. However, despite the matching efforts, a complete balance in age distribution between both groups could not be fully achieved, with the postmatching SMD of 0.41 (small to moderate effect size). This residual difference in age likely reflects the intrinsic differences in the age distribution of patients affected by hMPV and RSV.

Analysis from the matched cohorts revealed that hMPV patients received systemic corticosteroid therapy more frequently than those with RSV. This trend may be partly due to the empirical initiation of corticosteroids upon admission, often before the nasopharyngeal aspirate results for viral identification were available. Our results showed that the proportion of patients presenting with asthma exacerbations did not significantly differ between both groups. However, given the older age of children in the hMPV group, clinicians might have been more inclined to administer corticosteroids in the presence of wheezing, even without a diagnosis of asthma. Conversely, the younger age of children in the RSV group may have led to a more conservative approach in corticosteroid use. Additionally, the differences in corticosteroid usage could reflect the current understanding of their effectiveness in treating these infections. For RSV, the usage of corticosteroids has been shown to be not effective,^23^, ^24^ leading to their limited use in these cases. However, the role of corticosteroids for hMPV infections is less clear, which might account for their more frequent usage. Our observation of increased corticosteroid usage in the hMPV group aligns with findings of some studies^19^, ^20^ yet contrasts with others.^15^, ^25^ This highlights the variability and complexity in the treatment approaches for these respiratory infections.

Some studies have suggested that hMPV presents with less severe manifestations compared with RSV, evidenced by lower hospitalisation rates,^3^ reduced need for oxygen therapy^4^ and shorter hospital stays.^5^ However, our matched cohort analysis indicated comparable severity between both infections, which aligns with findings observed elsewhere.^7^, ^26^, ^27^ We observed similar proportion of patients requiring various forms of respiratory support, comparable duration of mechanical ventilation and oxygen therapy and no notable differences in overall hospital stay or PICU admission rates. These findings demonstrate that hMPV can be as severe as RSV infections, highlighting the importance of patient‐specific clinical management over the aetiology of the viral infection.

There are several limitations to our study. First, our study was conducted within a limited time frame, spanning just a few months. This short period of observation may limit the generalizability of our findings and could influence the observed mortality rates. A more extended study period could provide more representative data and strengthen our conclusions. Second, we utilised antigen detection methods for diagnosis, which are less sensitive than polymerase chain reaction (PCR)‐based techniques. This could result in underreporting of viral coinfections, potentially affecting the study outcomes. Additionally, the diagnostic methods we used were unable to distinguish between RSV subtypes A and B, and hMPV genotypes A and B, which have been previously associated with varying disease severities in previous studies.^25^, ^28^, ^29^ Third, the retrospective nature of our study could lead to incomplete data recording, and our analysis was limited by the lack of routine laboratory measures such as C‐reactive protein (CRP) levels and chest radiographs for all patients. These parameters could provide further insight into the clinical severity of the infections.

Our study demonstrated that hMPV and RSV are important respiratory pathogens causing hospitalisation in children. There were age demographic differences, with hMPV presenting at an older age. Despite these age variations, the clinical presentation of both viruses was similar, with the exception of fever, which was more prevalent among the hMPV patients. Both viruses demonstrate considerable utilisation of healthcare resources with similar rates of PICU admission. Our findings reinforce the need to treat both hMPV and RSV as equally significant causes of respiratory illness in the paediatric population.

## AUTHOR CONTRIBUTIONS

David Ng Chun‐Ern, Liew Chuin‐Hen and Tan Kah Kee conceived the study. David Ng Chun‐Ern, Liew Chuin‐Hen, Tan Kah Kee and Suhaila bt Baharuddin contributed to the design of the study. Elida Hanan binti Awang, Farah Nuruliayana binti Ahmad Nazri, Asuwani K. Tamil, Vishnu Arvindran a/l Chandra Mohan, Durairaaj Ramachandran, Chok Michelle, Teh Cheah Hooi and Airena Mohamad Nor made substantial contributions to data collection. Liew Chuin‐Hen analysed the data. David Ng Chun‐Ern, Liew Chuin‐Hen, Tan Kah Kee, Elida Hanan binti Awang, Farah Nuruliayana binti Ahmad Nazri, Asuwani K. Tamil, Vishnu Arvindran a/l Chandra Mohan, Durairaaj Ramachandran, Chok Michelle, Teh Cheah Hooi, Airena Mohamad Nor, Suhaila bt Baharuddin and Erwin Khoo Jiayuan contributed to data interpretation. David Ng Chun‐Ern, Liew Chuin‐Hen, Tan Kah Kee and Erwin Khoo Jiayuan wrote the first draft of the manuscript and supported literature review. David Ng Chun‐Ern, Liew Chuin‐Hen, Tan Kah Kee and Erwin Khoo Jiayuan revised the manuscript for important intellectual content. David Ng Chun‐Ern and Liew Chuin‐Hen produced the tables. David Ng Chun‐Ern and Erwin Khoo Jiayuan participated in obtaining funds and were involved in the process of securing funding acquisition. All authors reviewed the manuscript, giving approval for the final version to be published and agreed to be accountable for all aspects of their work.

## CONFLICT OF INTEREST STATEMENT

None.

## Supporting information


**Table S1:** Study Variables with Outlying Data.
**Table S2:** Study Variables with Missing Data.

## Data Availability

Data are available on reasonable request made to the corresponding author.
