# Supplementary material for: Clinical comparison of HMPV and RSV infections in hospitalised Malaysian children: A propensity score matched study
Source: Clin Respir J. 2024 Mar 26;18(3):e13747. doi: 10.1111/crj.13747 (PMC10964171; doi:10.1111/crj.13747)
Supplement: Supplementary file 1 — Table S1: Study Variables with Outlying Data. Table S2: Study Variables with Missing Data. [file CRJ-18-e13747-s001.docx]

**Supplementary Table 1: Study Variables with Outlying Data**

| Variables | Outlying values | Interventions |
| --- | --- | --- |
| Length of hospitalization, days | 2 (1%) | Natural outliers |
| Total white cell count, x 10^9^/l | 1 (0.5%) | Natural outlier |
| Platelet count, x 10^9^/ | 1 (0.5%) | Natural outliers |
| C-reactive protein, mg/dl | 2 (1%) | Natural outlier |
| Alkaline phosphatase, U/l | 2 (1%) | Natural outliers |
| Duration of oxygen therapy, days | 1 (0.5%) | Natural outliers |
| Day of illness on presentation, days | 9 (4.4%) | Natural outliers |

**Supplementary Table 2: Study Variables with Missing Data**

| Variables | Missing values | Interventions† |
| --- | --- | --- |
| Total white cell count | 24 (11.8%) | Multiple imputation |
| Platelet count | 24 (11.8%) | Multiple imputation |
| Absolute lymphocyte count | 25 (12.3%) | Multiple imputation |
| C-reactive protein | 118 (57.8%) | Delete variable from the analysis |
| Albumin | 172 (84.3%) | Delete variable from the analysis |
| Alanine transaminase | 175 (85.8%) | Delete variable from the analysis |
| Alkaline phosphatase | 175 (85.8%) | Delete variable from the analysis |

†For the multiple imputation process, we generated five imputed datasets to account for the uncertainty associated with missing data. We used the monotonic multiple imputation. We used linear regression to estimate missing values based on observed data from the covariates. Ultimately, only 190 rows (out of 192) had complete data after the imputations. Study variables with missing data in more than 50% of the cases were removed from the analysis.
